# Erectile dysfunction post-radical prostatectomy – a challenge for both patient and physician

**Published:** 2017

**Authors:** O Bratu, I Oprea, D Marcu, D Spinu, A Niculae, B Geavlete, D Mischianu

**Affiliations:** * Department of Urology, “Dr. Carol Davila” Central Military Universitary Emergency Hospital, Bucharest, Romania; ** Clinical Department No. 3, “Carol Davila” University of Medicine and Pharmacy Bucharest, Romania; *** Intensive Care Unit, “Dr. Carol Davila” Central Military Universitary Emergency Hospital Bucharest, Romania; **** Department of Nephrology and Dialysis, “Sf. Ioan” Clinical Emergency Hospital Bucharest, Romania; ***** Department of Urology, “Sf. Ioan” Clinical Emergency Hospital Bucharest, Romania

**Keywords:** erectile dysfunction, phosphodiesterase 5 inhibitors, vacuum erectile devices, intraurethral suppositories, penile implant prosthesis

## Abstract

Post-radical prostatectomy erectile dysfunction (post RP ED) is a major postoperative complication with a great impact on the quality of life of the patients. Until present, no proper algorithm or guideline based on the clinical trials has been established for the management of post RP ED.

According to literature, it is better to initiate a penile rehabilitation program as soon as possible after surgery than doing nothing, in order to prevent and limit the postoperative local hypoxygenation and fibrosis.

The results of numerous clinical trials regarding the effectiveness of the phosphodiesterase 5 inhibitors therapy on post RP ED have made them the gold standard treatment. Encouraging results have been achieved in studies with vacuum erectile devices, intraurethral suppositories with alprostadil and intracavernosal injections, but due to their side effects, especially in the cases of intracavernosal injections and intraurethral suppositories, their clinical use was limited therefore making them a second line option for the post RP ED treatment.

What should not be forgotten is that penile implant prosthesis has proven very effective, numerous studies confirming high rates of satisfaction for both patients and partners.

## Introduction

Due to the intense coverage of the prostatic specific antigen screening, prostate cancer has become one of the most frequently diagnosed cancers in western countries, over 90% of the patients being diagnosed in local or regional stages [**1**].

Radical prostatectomy in patients with localized prostatic cancer is one of the most commonly used therapeutic approaches. Although the oncologic long-term results are very good, the rate of postoperative erectile dysfunction must not be neglected, ranging between 14-90%, depending on the surgical approach and on the surgical experience [**2**-**4**].

The notion of erectile dysfunction can be defined as the inability to achieve and maintain an adequate erection for a satisfying sexual contact [**5**].

Taking into account the fact that the mean age of the patients diagnosed with prostatic cancer has decreased over the last decades, due to the early detection by using PSA screening, and that life expectancy has grown, we consider that the importance of postoperative erectile recovery has significantly increased, due to its impact on the patients’ quality of life as well as on their partners’.

Before performing radical prostatectomy, it is crucial to evaluate and establish each patient’s baseline erectile function, by using internationally validated psychometric instruments such as IIEF [**2**]. There are multiple factors that influence baseline erectile function, factors that should be taken into account when evaluating the postoperative erectile function recovery. It is known that age, diabetes, obesity, alcohol, smoking, chronic kidney disease, cardiovascular and neurological pathologies have an important role in the appearance of erectile dysfunctions [**6**-**8**]. It was demonstrated that the probability of early erectile function recovery after radical prostatectomy is higher for the patients with high preoperative IIEF scores when compared to the patients who already presented preoperative erectile dysfunction [**9**].

Another important factor involved in post-radical prostatectomy (RP) erectile dysfunction (ED) is the surgical factor. Surgical experience and technique have a crucial role in the appearance of post RP ED [**10**]. In patients with localized prostatic cancer who undergo RP or another major pelvic surgery, the risk of postoperatory ED is high due to neuropraxia [**4**]. Local inflammation and ischemia appear after a local trauma caused by cutting, coagulation, traction, compression of the pelvic structures for a better visualization of the operative field, thus affecting the cavernous nerves and resulting into reduced local oxygenation, pro-apoptotic and pro-fibrotic changes in corpora cavernosum, changes that are responsible for post RP ED [**2**,**3**].

The development of minimally invasive surgery allows a better visualization of the prostate and the periprostatic structures, a more precise dissection and excision, with lower intraoperative bleeding, therefore the rate of post RP ED is significantly lower for robot assisted radical prostatectomy (RARP) when compared to classical open surgery [**11**,**12**].

Cavernous nerve terminations originating from the pelvic plexus, located in the tissue between the rectum and the bladder, release nitric oxide during sexual stimulation that leads to an increased oxygenated blood flow to the erectile cavernous tissue and determines the relaxation of the smooth muscle fibers of the arteries and arterioles of the erectile tissue [**13**]. Nitric oxide is also produced by the endothelial cavernous cells due to vascular and sinusoidal forces on the endothelium [**2**]. The postoperative local hypoxia leads to a decrease in nitric oxide production and furthermore determines the inhibition of prostaglandin release (molecule that is strongly implicated in the inhibition of the pro-fibrotic process, inhibiting the accumulation of type I and III collagen in the smooth muscle fibers) with fibrotic tissue buildup, that in time will replace the cavernous smooth muscle fibers. The cavernous nerve fibers are accompanied by vascular structures and together form the neurovascular bundles [**2**].

Despite the surgical technique and approach used, various degrees of nerve damage will always exist, even in nerve sparing surgery, due to local trauma and its ischemic effect. The postoperative chronic penile flaccidity caused by neuropraxia leads to fibrotic and apoptotic changes in the erectile tissue, with vascular and cavernous elasticity impairment, changes that are responsible for the appearance of ED [**4**].

All things taken into consideration, postoperative penile rehabilitation should be introduced as soon as possible after surgery, to prevent fibrosis and to avoid irreversible structural changes that will determine end-organ damage responsible for the permanent ED. It has been demonstrated that any form of penile rehabilitation is better than doing nothing [**2**].

The notion of penile rehabilitation implies any form of treatment or combination of treatments that will lead to the recovery of the erectile function, similar to the preoperative baseline erectile function and furthermore to the ability of having a satisfying sexual intercourse [**14**].

In the first months after RP, patients will not be able to have spontaneous nocturnal erections despite the treatment applied. It is estimated that the recovery of a satisfactory sexual function will be achieved in 12 to 24 months after surgery with the help of a penile rehabilitation program [**15**].

The aim of the therapeutic approach used in post RP ED penile rehabilitation is to avoid and prevent structural endothelial and smooth muscle changes by improving the cavernous oxygenation. Therefore, the principle behind the majority of the penile rehabilitation therapies is early tissue oxygenation [**9**,**14**,**16**].

**Therapeutic approaches used in post RP ED**

The most common treatment used in post RP ED is the phosphodiesterase type 5 inhibitors (PDE5i) therapy, which is considered the first line treatment due to its ease of use, safe profile, and positive effect on the erectile function. This is followed by intracavernosal injections and vacuum erection devices and, the penile prosthesis implant, a solution with great results, regarded as a third line treatment for the patients with ED reluctant to medical treatment, should also be kept in mind.

**Phosphodiesterase 5 inhibitors**

Since their discovery in the late 90’s, due to their safe profile, ease of use and good response, the PDE5i therapy has changed and revolutionized the treatment of erectile dysfunction [**5**].

PDE5i enhance erectile function by decreasing the breakdown rate of cyclic guanosine monophosphate (cGMP), which leads to increased intracellular calcium ions efflux with smooth muscle relaxation and erection, this pathway being potentiated by nitric oxide via cavernous nerves [**17**-**19**].

PDE5i such as sildenafil, vardenafil, and tadalafil have been successfully used in patients with post RP ED.

In a randomized study conducted on 76 patients who have undergone bilateral nerve sparing open RP (BNSRP) and have received sildenafil nightly for 36 weeks or placebo after surgery, Padma Nathan demonstrated that the patients in the sildenafil group have shown increased IIEF-EF scores and improved nocturnal penile erections when compared to the placebo group [**20**,**21**].

In a study comparing the efficacy of a nightly 10 mg dose of vardenafil or on demand 10 mg vardenafil or placebo in patients after BNSRP, Montorsi showed that the results with the PDE5i treatment were superior to those with placebo in both groups of vardenafil patients, but it also showed no difference between the two vardenafil groups after a period of two months drug washout [**22**].

In a 2014 randomized placebo controlled study (REACTT), Montorsi evaluated the efficacy of tadalafil on a group of 423 patients who have undergone NSRP. The 423 lot was divided randomly in three groups and the patients have received 5 mg tadalafil (139) once daily, 20 mg tadalafil (143) on demand and placebo (141). After nine months of treatment, the IIEF- scores have improved in both tadalafil groups, but when compared to the placebo group the results were significantly higher in the daily tadalafil group. After a six weeks drug free period, no significant differences between the groups were seen. At the end of the study, it was also concluded that the daily use of tadalafil has had an important role in preventing penile length loss [**23**].

Avanafil, another PDE5i that was recently released, has shown significant improvements in treating patients with post RP ED, especially those who failed to respond to the treatment with sildenafil, vardenafil, or tadalafil.

In a 2013 phase three double blind placebo controlled study regarding the safety and efficacy of avanafil in the treatment of RP ED, Mulhall randomized 298 patients to 100 mg or 200 mg of avanafil or on demand placebo for 12 weeks. Prior to the start of the study, the patients presented erectile dysfunction for six weeks or more, after RP was performed. At the end of the study it was concluded that the avanafil treatment was superior to placebo, with a significant increase of the IIEF score as well as in the Sexual Encounter Profile-question 3 (Did your erection last long enough for you to have a successful intercourse?) [**24**,**25**].

Daily chronic administration of PDE5i has proven to be the first choice in the treatment of post RP ED. The debate between the efficacies of daily use of PDE5i versus on demand PDE5i is still open. Until now, no clinical trial has demonstrated the superiority of a daily administration of PDE5i versus on demand administration [**20**]. The majority of the clinical trials have demonstrated that the positive effects of the PDE5i regarding the penile rehabilitation program do not seem to persist after a drug free period.

According to literature and keeping in mind the results of the existing clinical studies, it is undoubtedly better to use a PDE5i therapy in post RP penile rehabilitation programs than to neglect the postoperative recovery.

**Vacuum erection devices (VED)** function by creating a vacuum around the penis and drawing blood into corpus cavernosum by using a manually pump which creates a negative pressure. A constriction ring can be applied at the base of the penis to prevent the blood outflow, therefore sustaining the erection [**2**,**9**].

This is a safe, cheap and with good results therapeutic method for erectile dysfunction, therefore its popularity has grown. Its ability to restore the long term erectile function and to avoid the irreversible fibrotic changes in the corpus cavernosum is questionable, due to the fact that the erection produced by the vacuum devices consists in both arterial and venous blood, in variable proportions, and that the oxygen saturation is around 76%, not enough to avoid the fibrotic and pro-apoptotic changes [**2**]. There are studies that are in favor of the vacuum erection devices, especially when they are associated with PDE5i.

In a prospective clinical trial conducted on 109 patients regarding the early use of vacuum erectile devices following RP, Raina concluded after 9 months that 80% of the patients using VED have had erections strong enough for a satisfying sexual intercourse and that they were less likely to present penile shrinkage [**17**,**26**].

Another study conducted by Basal on 203 patients who have used VED, PDE5i, VED combined with PDE5i or no treatment after RP has demonstrated that only PDE5i or the association of PDE5i with VED have had a positive effect regarding the postoperative erectile function recovery [**27**].

Due to their simple and early use after RP, low price, safe profile and the fact that they can ensure multiple daily erections, the VED are preferred by numerous patients, but their effect regarding the long term erectile recovery is questionable.

**Intraurethral therapy with Alprostadil suppository** (a PGE1 analog) has proven to have a positive effect on the IIEF scores. PGE1 acts by increasing the cAMP level and oxygenation by promoting blood flow [**2**].

In a study evaluating the efficacy of intraurethral alprostadil suppositories (IUA) versus nightly sildenafil conducted on 212 patients, McCullough reported no statistically significant differences regarding intercourse success and IIEF scores between the two groups after nine months of treatment. A significant difference in favor of the IUA has been seen at six months, but this could have been be due to the fact that neuropraxia still existed at six months after surgery and that the PDE5i treatment was not fully effective. The compliance rate was in favor of the sildenafil treatment (98% vs. 79%), due to the local pain induced by IUA [**28**].

Although IUA therapy can have significant results in the postoperative erectile recovery, the lack of clinical trials and as well as the high price and side effects (urethral burning and penile pain) make its use limited in the medical community [**17**].

**Intracavernosal injections (ICI)** represent a form of therapy that can be used in patients who have tried PDE5i and have failed [**9**]. The first study regarding the ICI was realized by Montorsi in 1997 on 30 patients who were randomized to receive ICI with alprostadil three times a week for three months versus no treatment. After six months he concluded that 67% of the patients from the ICI group achieved spontaneous erections, strong enough for a satisfying intercourse [**17**,**29**].

Mulhall enhanced the role of ICI therapy as an alternative to PDE5i in two studies, showing that ICI is a feasible option for erectile function recovery [**25**,**30**].

Alprostadil ICI can induce penile pain that may lead to treatment dropout, but the TriMix ICI (papaverine, phentolamine, and PGE1) is associated with less pain and therefore it can be well supported by patients [**2**].

Papaverine is a PDEi that can increase cAMP and cGMP levels in erectile tissue and phentolamine is an alpha-blocker that determines smooth muscle relaxation. The combination of these three molecules (papaverine, phentolamine, and PGE1) acts as a vasoactive agent increasing the corpus cavernosum blood flow and determining erections and penile engorgement [**17**].

**Penile prosthesis implantation** represents the third line treatment for the post-radical prostatectomy erectile dysfunction. Since its introduction in the 1970s, it has been improved and due to the evolution of the surgical techniques, it has become an effective treatment for erectile dysfunction, with excellent efficacy and satisfaction rates for both patients and their partners [**5**].

Megas compared the efficacy and satisfaction profile between the PDE5 inhibitors therapy and penile prosthesis implantation on a lot of 54 patients who have undergone nerve sparing RP. The patients included in the study were at six months after RP, were disease free (prostatic cancer) and all of them suffered from erectile dysfunction. The lot was divided into two groups, one receiving PDE 5 inhibitors and the patients in the second group have undergone penile prosthesis implantation. The patients were evaluated before surgery and at 6, 12, and 24 months after surgery. Both groups have encountered improvements regarding the quality of erection and in the ability to have a successful sexual contact, but the results were better for the patients in the penile prosthesis group [**31**,**32**].

There are numerous studies with great results regarding the efficacy, safety, and satisfaction of the penile prosthesis. It was proven that the penile prosthesis option in the treatment of post RP ED is superior to the PDE5 inhibitors therapy, especially when evaluating parameters such as penile firmness, penetration ability, intercourse frequency, patient confidence, and satisfaction [**3**].

**Low intensity ESWT**

The principle behind LI-ESWT in the treatment of post RP ED is that it induces local microtrauma with biological changes that lead to neovascularization, thus improving the smooth muscle cells and the endothelial cells, with an up-regulation of the VEGF, neuronal NO synthase and von Willebrand factor [**33**-**35**].

Frey conducted a small study on 16 patients with ED after robot assisted RP. The patients received six sessions of LI-ESWT during a period of six weeks and they were evaluated at one month and at 12 months after treatment. He concluded that the IIEF 5 scores increased with 3,5 points at one month and with one point at 12 months [**36**].

Other studies regarding the efficacy of LI-ESWT in ED have reached similar encouraging conclusions, but the lack of large randomized clinical studies makes this option not so popular in the urological community [**37**].

Experimental and innovative therapeutic approaches have lately emerged with interesting results, but the lack of large studies that validate their effectiveness and safety make them not usable in the clinical practice. The use of options such as stem cells (adipose tissue derived SC, bone marrow SC, human umbilical cord SC), combined SC with LI-ESWT, impulse magnetic field therapy, nanotechnology, tissue engineering and endovascular tools should not be excluded in the future [**35**,**38**-**40**].

## Discussions

The penile rehabilitation program is based on improving cavernosal oxygenation and preventing endothelial and smooth muscle irreversible fibrotic changes. It has been proven that it is better to start a penile rehabilitation program as soon as possible after surgery, to limit the fibrotic changes that lead to ED.

Despite the existence of various therapeutic alternatives for treating ED, a proper algorithm or a clinical guideline that could enlighten us regarding the proper management of the post RP ED has not been established until now.

Moskovic elaborated a penile rehabilitation program by combining the PDE-5 inhibitors, intraurethral alprostadil suppositories or intracavernosal injections and vacuum erection devices. The patient is advised to start the program a week before surgery with a nightly dose of 25 mg sildenafil and three times a week intraurethral 250 μg alprostadil suppositories. Three days after surgery the nightly PDE-5 inhibitor is restarted and the alprostadil intraurethral suppositories are added after the urethrovesical catheter removal (three times a week). The patients are evaluated one month after surgery. The dose of alprostadil is titrated if necessary and all the patients are recommended to use VED for minimum 10 minutes a day. If the results are not satisfactory after three months of treatment the alprostadil suppositories will be changed with intracavernosal injections with TriMix (three times a week) and the patients will be reevaluated monthly in order to see if the alprostadil suppositories could be reintroduced. The patients are evaluated for almost two years and in the cases of non-responders, penile prosthesis implantation is recommended [**2**,**41**].

## Conclusions

With the early detection of prostatic cancer and curable surgery, the postoperative erectile recovery has become an important goal. Post RP ED represents a challenge for both patient and physician, with an important impact on the quality of life.

The incidence of post RP ED remains high despite the surgical approach and it represents a serious problem, especially for young patients. It has been demonstrated that robot assisted RP has a lower incidence for postoperative ED when compared to the results encountered in patients who have undergone laparoscopic or open RP.

A proper algorithm or a clinical guideline for the post RP ED has not been established until now. It has been demonstrated that the early start of a penile rehabilitation program has significantly reduced the postoperative erectile recovery period, as well as it has reduced the irreversible postoperative local fibrosis and even improved the baseline EF when compared to the patients who have not been included in a penile rehabilitation program.

The effectiveness of the penile rehabilitation program depends on the patient’s age, comorbidities, preoperative baseline EF, surgical approach, surgeon’s experience, and compliance to the treatment. An important aspect for both patient and physician is that the preoperative baseline EF is hard to recover, thus it is crucial that the patient should be informed about this risk before surgery.

The PDE-5 inhibitors represent the first line treatment in post RP ED, numerous clinical trials having proven their safety and potency. Vacuum erection devices, intraurethral suppositories with alprostadil and intracavernosal injections represent an alternative for the patients who have failed to respond to the PDE-5 inhibitors, but with a low compliance rate especially for the intracavernosal injections and intraurethral suppositories due to their side effects. The penile prosthesis implant represents the final solution for the patients reluctant to medical treatment, with great results regarding safety and patients’ satisfaction.

**Acknowledgement**

This paper is supported by project “Optimizarea diagnosticului si tratamentului pacientilor cu disfunctie erectile” (Optimizing the diagnosis and treatment of patients with erectile dysfunction), Contract no. 34659/ 02.11.2015.
